# The CD27L and CTP1L Endolysins Targeting *Clostridia* Contain a Built-in Trigger and Release Factor

**DOI:** 10.1371/journal.ppat.1004228

**Published:** 2014-07-24

**Authors:** Matthew Dunne, Haydyn D. T. Mertens, Vasiliki Garefalaki, Cy M. Jeffries, Andrew Thompson, Edward A. Lemke, Dmitri I. Svergun, Melinda J. Mayer, Arjan Narbad, Rob Meijers

**Affiliations:** 1 European Molecular Biology Laboratory (EMBL), Hamburg, Germany; 2 Synchrotron Soleil, L'Orme des Merisiers, Saint Aubin, Gif sur Yvette, France; 3 European Molecular Biology Laboratory (EMBL), Heidelberg, Germany; 4 Institute of Food Research, Colney, Norwich, United Kingdom; National Jewish Medical and Research Center, United States of America

## Abstract

The bacteriophage ΦCD27 is capable of lysing *Clostridium difficile*, a pathogenic bacterium that is a major cause for nosocomial infection. A recombinant CD27L endolysin lyses *C. difficile* in vitro, and represents a promising alternative as a bactericide. To better understand the lysis mechanism, we have determined the crystal structure of an autoproteolytic fragment of the CD27L endolysin. The structure covers the C-terminal domain of the endolysin, and represents a novel fold that is identified in a number of lysins that target Clostridia bacteria. The structure indicates endolysin cleavage occurs at the stem of the linker connecting the catalytic domain with the C-terminal domain. We also solved the crystal structure of the C-terminal domain of a slow cleaving mutant of the CTP1L endolysin that targets *C. tyrobutyricum*. Two distinct dimerization modes are observed in the crystal structures for both endolysins, despite a sequence identity of only 22% between the domains. The dimers are validated to be present for the full length protein in solution by right angle light scattering, small angle X-ray scattering and cross-linking experiments using the cross-linking amino acid p-benzoyl-L-phenylalanine (pBpa). Mutagenesis on residues contributing to the dimer interfaces indicates that there is a link between the dimerization modes and the autocleavage mechanism. We show that for the CTP1L endolysin, there is a reduction in lysis efficiency that is proportional to the cleavage efficiency. We propose a model for endolysin triggering, where the extended dimer presents the inactive state, and a switch to the side-by-side dimer triggers the cleavage of the C-terminal domain. This leads to the release of the catalytic portion of the endolysin, enabling the efficient digestion of the bacterial cell wall.

## Author Summary

*Clostridium difficile* infection is a common cause of hospital-acquired diarrhea, following broad-spectrum antibiotic treatment particularly in elderly patients. Bacteriophage therapy could provide an alternative treatment, but a better understanding of the viral components that lyse the bacterial cell is necessary. Here, we report on the activation of two endolysins from bacteriophages that lyse *Clostridia*. The structures of autoproteolytic fragments of two endolysins were determined by X-ray crystallography. Based on the structures, we introduced mutations that affect the autolytic cleavage of the enzymatic portion of the endolysins, and we show that two oligomeric states have an effect on the cleavage mechanism. Moreover, the lysis activity is affected when autocleavage is inhibited for one endolysin. We propose that the cleavage and oligomerization are linked, and they provide the endolysin with a trigger and release mechanism that leads to activation. The identification of a trigger and release factor may not only be relevant to *Clostridia* endolysins, but could be an important factor in the triggering of many bacteriophage endolysins. A fuller understanding of this activation mechanism will help in the design of recombinant endolysins or bacteriophages with a more efficient therapeutic potential.

## Introduction

The increasing emergence of antibiotic resistance has led to a renaissance in the use of bacteriophage therapy as an alternative to eradicate pathogenic bacteria [1]. These bacterial viruses are potentially effective bactericides, with the additional advantage that they only affect a small portion of the human microbiome, in contrast to the broad spectrum antibiotics in use [2], [3]. Many antibiotics have an effect on a large portion of the microbiome, leading to a shift in bacterial populations after treatment. A striking example is the emergence of *Clostridium difficile* as a causative agent of antibiotic-associated diarrhea. *C. difficile* is resistant to many of the antibiotics used in hospitals, and it colonizes the gut after antibiotic treatment [4]. In search of an alternative treatment, a bacteriophage named ΦCD27 was isolated from a strain of *C. difficile* [5]. The genome of the ΦCD27 phage revealed the presence of a canonical holin/endolysin system. Endolysins are produced by many double stranded DNA bacteriophages to effect the release of new virions from an infected cell by degrading the bacterial cell wall [6]. The recombinant endolysin CD27L was shown to lyse *C. difficile in vitro* [5]. We have also shown that the N-terminal domain of CD27L consisting of a zinc dependent N-acetylmuramoyl-L-alanine amidase alone is effective in lysis, and that the host range of the endolysin can be affected by a mutation in the substrate binding pocket [7].

Bacteriophages co-evolve with their bacterial hosts, and the continuous adaptation of the phage may limit its lethality. Many bacteriophages isolated from the host environment are therefore not efficient in the rapid eradication of pathogenic hosts, as is the case with ΦCD27. The potential to engineer more potent bacteriophages requires knowledge of the most important components of the lysis machinery [8]. Cell lysis is tightly regulated by the phage which only triggers cell lysis once it has finished the production of new viral particles inside the cell [9]. The endolysin is sequestered in the cytoplasm until it can penetrate the peptidoglycan layer following the formation of lesions in the cell membrane that are created by holin, another phage encoded protein [10]. Endolysins typically consist of a peptidoglycan hydrolase domain and a C-terminal domain that is often termed as a cell wall binding domain. The efficient use of endolysins as bactericides is limited by a poor understanding in most systems of the mechanisms that relate catalytic activity to the role of the C-terminal domain [8]. Many recombinantly produced endolysins can lyse a population of bacteria efficiently only after the protein has been incubated or converted with cell wall material from the host [11], [12]. For some endolysins, the catalytic domain expressed in isolation is more effective than the full-length protein [7], [13], and for other endolysins, the catalytic domain alone shows reduced or no lytic activity at all [12], [14]. For a pinholin-dependent phage, endolysin activation was shown to depend on disulphide isomerisation that triggers cleavage of the enzyme from the bacterial membrane [15]. For the highly efficient endolysin PlyC active against streptococcal species, it was found that two catalytic components are tethered in a non-covalent way to eight components of the cell wall binding domain [16]. However, for the classical endolysin/holin system, it is not clear how the endolysins are activated. Here, we present the crystal structures of autoproteolytic fragments of the CD27L and CTP1L endolysins, covering the C-terminal domain. Structure-based mutagenesis allowed us to manipulate autolytic cleavage, and we show that the rate of cleavage is proportional to lysis efficiency for the CTP1L endolysin.

## Results

### The C-terminal domain of CD27L adopts a novel protein fold

When crystallization trials for full length CD27L endolysin were set up, crystals appeared overnight from freshly purified protein. Any delay in the purification or crystal tray setup would prevent crystallization, and the crystals dissolved after three weeks. An X-ray data set to 2.3 Ångstrom was collected from a fresh crystal, and it was realized that the crystal most likely contained the C-terminal portion of the endolysin, because molecular replacement with the previously determined crystal structure of the catalytic domain [7] was not successful. To determine the structure, the C-terminal portion of CD27L was also independently cloned, expressed and purified. This N-terminal deletion construct (N-CD27L) was crystallized, and the crystal structure was determined by single wavelength anomalous diffraction (SAD) using a mercury derivative (See Table 1 for details). The structure was used as a model to solve the structure of the full-length CD27L crystals by molecular replacement. It was found that the “full length” construct had been proteolyzed and the crystal contained six copies of the C-terminal portion of CD27L alone. The refined structure shows clear electron density for all six monomers of the C-terminal domain with a Matthews coefficient of 2.3, and there is no space in the crystal lattice for an additional N-terminal domain.

**Table 1:** Data collection and refinement statistics.

	CD27L Proteolytic fragment	CTP1L V195P mutant (proteolytic fragment)#	ΔN-CD27LHg derivative
**Data collection**			
Space group	P2_1_2_1_2_1_	I222	P2_1_
Cell dimensions			
*a*, *b*, *c* (Å)	75.3, 82.1, 83.8	44.9, 48.8, 77.2	63.1, 84.7, 65.3
α, β, γ (°)	90.0, 90.0, 90.0	90.0, 90.0, 90.0	90.0, 92.0, 90.0
Wavelength (Å)	0.970	1.223	0.998
Resolution (Å)	20–2.24 (2.37–2.24)*	20–2.10 (2.17–2.10)*	20–3.5 (3.66–3.50)*
*R*_sym_ or *R*_merge_	12.8 (59.8)*	2.5 (4.4)*	23.8 (55.8)*
*I*/σ*I*	7.0 (2.0)*	48.9 (29.0)*	4.7 (2.6)*
Completeness (%)	97.9 (93.8)*	92 (55.5)*	99.9 (99.9)*
Redundancy	2.7 (2.6)*	5.7 (4.4)*	4.8 (4.8)*
**Refinement**			
Resolution (Å)	30–2.24	20–2.10	
No. reflections	24189	4489	
*R*_work_/*R*_free_	18.9/24.7	17.2/26.4	
No. atoms			
Protein	4052	645	
Ligand/ion	n/a	n/a	
Water	398	90	
*B*-factors			
Protein	45	22	
Ligand/ion	n/a	n/a	
Water	57	32	
R.m.s. deviations			
Bond lengths (Å)	0.01	0.01	
Bond angles (°)	1.3	1.0	

*Values in parentheses are for highest-resolution shell.

# The data collection was affected by ice rings and a limited detector geometry.

**Figure 1:**
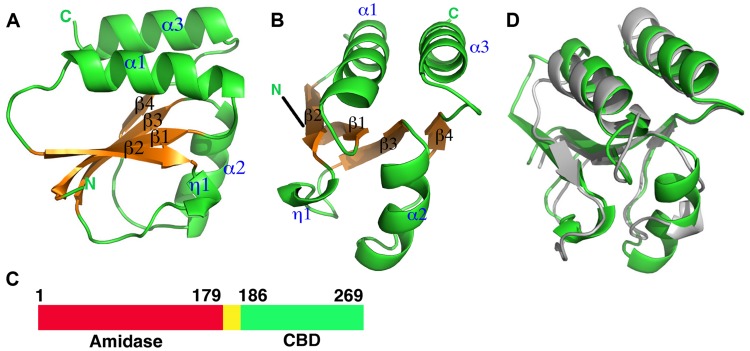
Overall structures of the proteolytic fragments of the CD27L and CTP1L endolysins reveal a novel fold for the C-terminal domain. A. Ribbon diagram of the C-terminal domain of endolysin CD27L. The beta sheet at the core of the domain is colored in gold and the secondary structure elements have been labeled. B. View of the C-terminal domain at a 90 degree angle from the view presented in A. C. Linear map of the domain organization of CD27L, with the enzymatic portion (Amidase) in red, the linker in yellow and the C-terminal domain in green. D. Superposition of the ribbon diagrams of the C-terminal domains of CTP1L (gray) and CD27L (green). Molecular graphics were produced with Pymol (The PyMOL Molecular Graphics System, Version 1.5.0.4 Schrödinger, LLC.).

**Figure 2:**
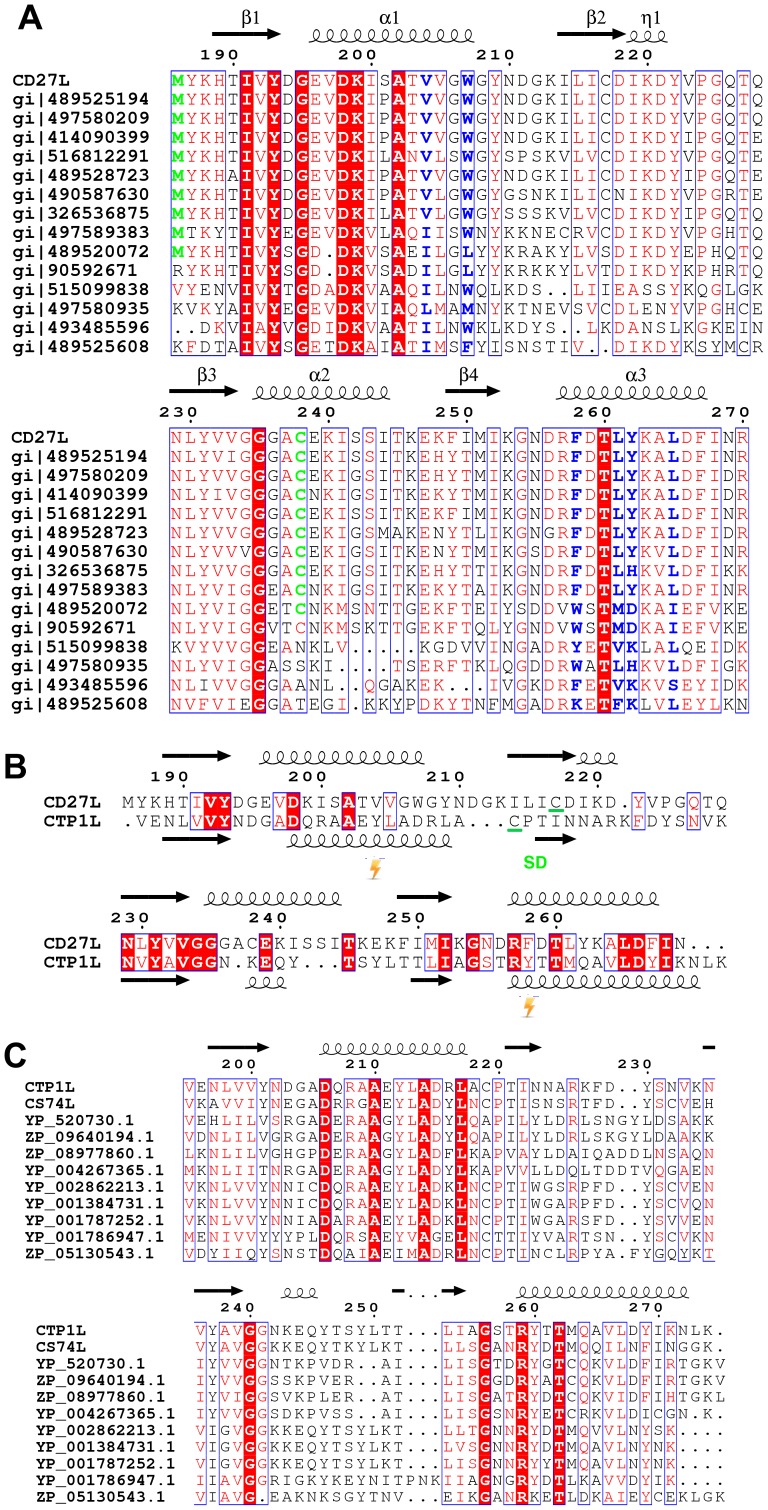
Sequence alignment of the C-terminal domains of CD27L and CTP1L showing this fold is prevalent among lysins that target *Clostridia*. A. Sequence alignment of the C-terminal domain of CD27L and other sequences with a significant BLAST score (E<0.01) produced with ESPRIPT [49]. Conserved residues are colored red. The secondary structure of the C-terminal domain of CD27L is depicted with arrows for beta strands and curls for alpha helices. Hydrophobic residues that contribute to the head-on dimer interface are colored blue, and the cysteine residue involved in the side-by-side dimer formation is colored green. B. Structure-based sequence alignment of the C-terminal domain of CD27L with the C-terminal domain of CTP1L. Residues where the cross-linking amino acid pBpa is inserted are marked by a blitz, and the area involved in the side-by-side dimer is marked SD. C. Sequence alignment of the C-terminal domain of CTP1L with other sequences that give a significant BLAST score. All sequences listed from the BLAST analysis are annotated as Clostridial lysins or endolysins.

The C-terminal portion of CD27L consists of a platform of four parallel beta strands, flanked by an alpha helix with two additional alpha helices mounted on top (Figure 1A,B). The N-terminus contains beta strand β1 at the center of the beta sheet, connected to alpha helix α1. This is followed by beta strand β2 at the outer side of the sheet that is connected through an extended loop including a single 3^10^ helical turn (η1) to beta strand β3 at the center of the beta sheet. The α2 alpha helix connects beta strands β3 and β4 and the fold ends with an alpha helix α3 at the C-terminus of the protein. A DALI search of the PDB for domains with a similar fold did not identify a structure with significant similarity [17]. A BLAST search was done with the sequence covering the proteolytic fragment to identify other proteins that may have a similar domain, and 14 unique sequences were found with an E value <0.01. All these proteins are lysins that target *Clostridia* species, and the sequence variation is too large to identify residues that define the fold (Figure 2A).

### Proteolytic processing of full length recombinant endolysin CD27L

**Figure 3:**
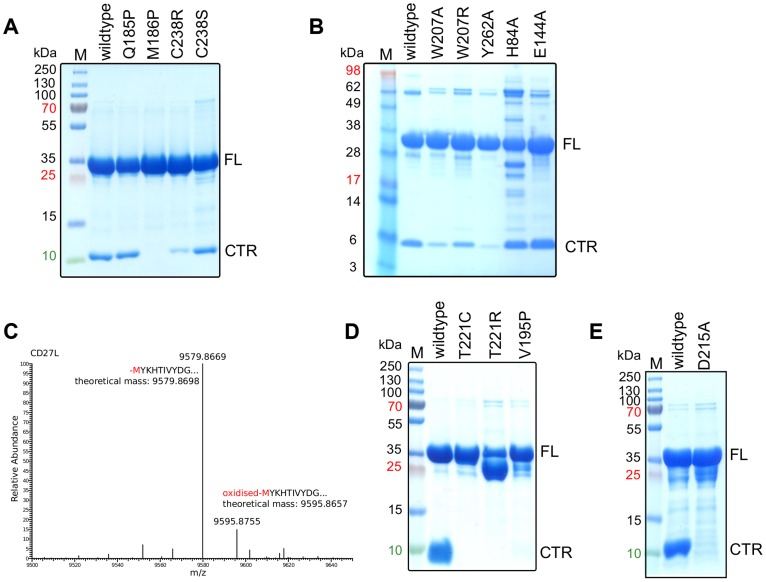
The cleavage of the CD27L and CTP1L endolysins occurs at a specific site and is affected by mutagenesis. A, B. SDS-PAGE gel of equal amounts of purified samples of CD27L wild-type and mutants, showing the full length (FL) protein and the 10 kDa band that corresponds to the C-terminal domain (CTR). C. LC-MS spectrum of the eluted fraction corresponding to C-terminal domain of CD27L, showing that the proteolysis occurs at M186. D, E. SDS-PAGE gel of equal amounts of purified wild-type and mutants of CTP1L. The mutants V195P, T221C, T221R and D215A inhibit autocleavage.

Expression of the full-length CD27L endolysin was hampered by severe and continuous proteolysis that could not be diminished by protease inhibitors. An SDS-PAGE gel of freshly purified material typically showed a protein band for the full length protein and a second band with a molecular weight that corresponds to the C-terminal domain (Figure 3A, B). Proteolytic products isolated from an SDS-PAGE gel of CD27L were analyzed by mass spectrometry following tryptic digestion, and this confirmed that the fragments were the intact N-terminal catalytic domain and the C-terminal domain respectively. The proteolytic fragment covering the C-terminal domain was also observed in liquid chromatography coupled to an electrospray mass spectrometry system, and the N-terminal residue was identified as methionine M186 (Figure 3C).

These observations are not unprecedented, and similar proteolytic processes can be uncovered from studies on other unrelated endolysins. For instance, crystallization of several endolysins was achieved only after a substantial incubation period [11], [18], or the individual domains had to be cloned and crystallized separately due to the degradation of the full-length protein [13]. By investigating the structures of full-length endolysins that underwent these treatments (PDB codes 1XOV [18] and 2IXU [19]) we observed that the linker between the domains is always extended and exposed to the solvent. In addition, the catalytic domain and the C-terminal domain are expressed as separate components in PlyC, the most efficient endolysin isolated to date [16]. This raised the possibility that the autoproteolytic cleavage of the catalytic domain in CD27L has a functional role.

### Mutagenesis at the cleavage site prevents endolysin cleavage

In an attempt to find the residues involved in the cleavage of the endolysin, we investigated the N-terminus of the proteolytic fragment of CD27L. The catalytic domain precedes the C-terminal domain, and when the two crystal structures are concatenated, there is a seven residue linker between the domains (Figure 1C). The autolytic fragment of the C-terminal domain starts at the end of the linker at methionine M186, which is still integrated in the C-terminal domain. Among the six copies of the C-terminal domain, there are no consistent contacts between M186 and other residues within the C-terminal domain. The methionine side chain only forms a hydrogen bond with the main chain nitrogen of threonine T227 in two out of six molecules.

Since there is no clear candidate among the adjacent residues to be involved in protein cleavage, we decided to mutate methionine M186 to a proline. The M186P mutant will strengthen the main chain at the cleavage point, and would alter the mechanics of the linker at the hinge close to the C-terminal domain. Indeed, the M186P mutant abolishes the cleavage of the endolysin as observed by SDS-PAGE (Figure 3A). In addition, we mutated the amino acid that precedes the methionine (glutamine Q185) to a proline. This residue forms part of the linker and is fully exposed to the solvent. In this case, endolysin cleavage was not affected. This indicates methionine M186 is critical in the cleavage process, and it forms an integral part of the C-terminal domain that is not accessible for external proteolytic cleavage.

### Structure of the C-terminal domain of the CTP1L endolysin mutant V195P

Another previously characterized phage endolysin that targets Clostridia is CTP1L, which lyses *C. tyrobutyricum* [14]. This endolysin also contains a C-terminal domain that is approximately 80 residues long, but the sequence identity with the C-terminal domain of CD27L is low (22%). SDS-PAGE analysis confirmed that purified CTP1L wild type endolysin undergoes cleavage of the C-terminal domain (Figure 3D). We then transferred the critical mutation that affected CD27L cleavage, involving the stem of the linker of the C-terminal domain of CTP1L (V195P). The SDS-PAGE analysis of purified recombinant CTP1L shows a reduction in cleavage for the V195P mutant (Figure 3D).

We attempted to crystallize the CTP1L V195P mutant to see if this slowly cleaving mutant would yield crystals of the full length protein. After 2 weeks, crystals appeared, an X-ray data set was collected to 2.1 Ångstrom and the structure of the C-terminal domain was solved by molecular replacement using the C-terminal domain of CD27L as a search model. As with CD27L, there was no N-terminal domain present in the crystal lattice. The C-terminal domain is truncated at Pro195, and there is only one molecule present in the asymmetric unit. The fold of the C-terminal domain of CTP1L is very similar to that of CD27L, except that the second alpha helix α2 is deleted, and the alpha helix α3 is extended in CTP1L (Figure 2B). A superimposition of the two domains based on secondary structure elements using Coot [20] gives an RMSD of 1.5 Å for 75 aligned residues, even though the domains have a low sequence identity (Figure 1D). A BLAST search for other proteins that align to the C-terminal domain of CTP1L reveal a separate set of amino acid sequences of lysins targeting *Clostridia* (Figure 2C). It is not possible to come up with conserved amino acids that define the fold. The only conserved residues are an aspartate on helix α1 (Asp 206 in CTP1L and Asp198 in CD27L), a threonine on helix α3 (Thr 262 in CTP1L and Thr 261 in CD27L), and an arginine (Arg 259 in CTP1L and Arg 258 in CD27L). The conserved aspartate/threonine form a hydrogen bond through a water molecule in both structures, connecting the outer alpha helices, but this is not sufficient to keep the fold together.

### Two dimerization modes suggestive of endolysin activation

**Figure 4:**
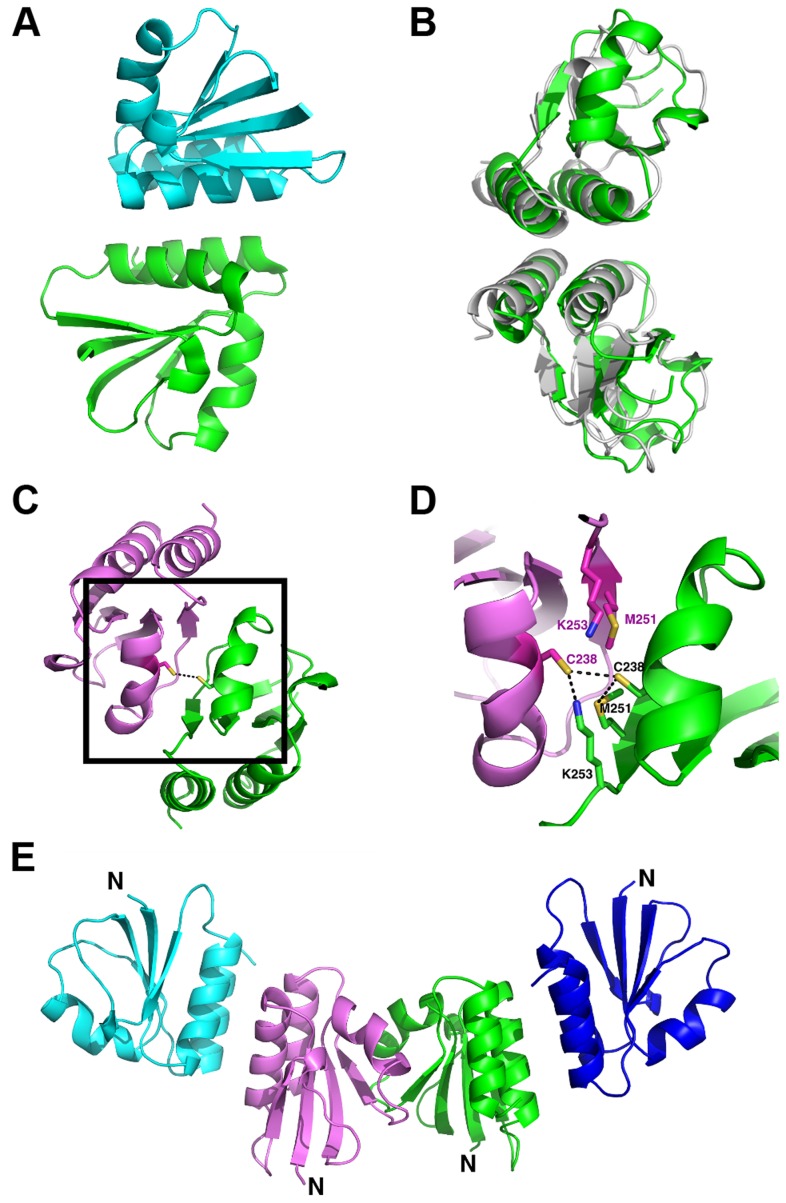
Overview of the oligomerization modes observed in the crystal structure of the proteolytic fragment of endolysin CD27L. A. Ribbon diagram of the head-on dimer configuration with one monomer coloured green and the other blue. B. The head-on dimer configuration rotated 90 degrees along the horizontal axis, with the head-on dimer of CTP1L superimposed in gray. C. Ribbon diagram of the side-by-side dimer, with one monomer in green and the other in pink. The side chain of cysteine C238 is shown as ball and stick for both monomers. D. Close-up of the side-by-side dimer interface, showing the interactions between cysteine C238 and symmetry mates of lysine K251 and methionine M253 shown in sticks. E. Tetrameric assembly observed in the crystal structure, with the side-by-side dimer in the center, where the N-terminus points in the same direction for both monomers, and two head-on dimers at the periphery.

The proteolytic fragments of CD27L form a mixture of dimers within the crystal lattice. All six molecules are engaged in one common dimerization mode, where the alpha helices α1 and α3 from one molecule stack on their symmetry mate from a second molecule. The α1 and α3 helices run parallel and in the same direction, forming a platform with a concave surface (Figure 4A). The dimerization is such that the N-termini of both monomers are pointing away from the dimer interface, and we term this dimerization mode a ‘head-on’ dimer. The buried surface area is between 1200 and 1300 Å^2^ for the three head-on dimers found in the asymmetric unit, as determined by the PISA server [21]. The docking for the three head-on dimers observed in the crystal lattice is very similar, and superimposition of the Cα atoms with LSQKAB [22] using both protomers gave RMSDs of 0.71 Å and 0.84 Å respectively. There is a 2-fold symmetry axis running perpendicular to the parallel alpha helices, with a hydrophobic core at the center consisting of residues valine V204, leucine L261 and leucine L265. Further along the rim, there are additional aromatic residues (tryptophan W207, phenylalanine F258 and tyrosine Y262) whose symmetry mates are involved in dimerization. The strong hydrophobic component, combined with the stacking of aromatic rings indicates this is a stable dimerization mode.

The head-on dimer is also present in the crystal lattice of the C-terminal domain of CTP1L. In fact, it is possible to superimpose the whole dimer unit based on secondary structure elements in Coot, with an RMSD for the Ca backbone of 2.1 A for 146 residues out of a total of 160 (Figure 4B). None of the residues in the head-on dimer face is conserved between CTP1L and CD27L. To test the significance of the head-on dimer, we performed mutagenesis on two of the aromatic residues involved in the dimer interface of CD27L (W207A/W207R and Y262A), as well as an aspartic acid situated at the edge of this dimer in CTP1L (D215A). These mutants had a surprising effect on the autolytic cleavage, since a decrease was observed in the cleavage product present on an SDS-PAGE gel (Figure 3B, E). These mutants are situated at the opposite site of the linker that connects the C-terminal domain to the catalytic domain.

An alternative dimerization mode is found among the six C-terminal domains present in the crystal structure of the proteolytic fragment of CD27L endolysin, between two molecules that are each involved in separate head-on dimers as well (Figure 4C). The α2 helices of the opposing monomers stack against each other and the buried surface area is 1216 Å^2^, similar to the values found for the head-on dimer. The side chains of cysteine C238 of the symmetry mates face each other at the center of this dimer, with a sulphur-sulphur distance of 3.4 Å (Figure 4D). This distance is too large to qualify for a covalent bond. The cysteine is in close proximity to methionine M251 (Figure 4D), with a sulphur-sulphur distance of 3.7 Å (4.1 Å for the symmetry mate). Moreover, lysine K253 forms a hydrogen bond between the NZ atom and the sulphur with a distance of 2.8 Å (3.1 Å for the symmetry mate). Together, M251 and L253 seem to destabilize the formation of the disulphide bond. Although the closely related phage endolysins contain cysteine C238 (Figure 2A), the surrounding residues seem to vary.

To test the significance of this side-by-side dimer, we mutated cysteine C238 to a serine (C238S) or an arginine (C238R), eliminating a potential disulphide bond. The arginine mutant had the strongest effect on the autoproteolytic cleavage, similar to the head-on dimer mutant (W207A), showing a significant reduction in the production of the proteolytic product (Figure 3A). The cleavage site M186 is approximately 20 Ångstrom away from cysteine C238, indicating that disruption of both dimer interfaces have an effect on the autocleavage of the endolysin.

There is a similar side-by-side dimer present in the crystal lattice of the CTP1L C-terminal fragment, but the domains point in opposite directions compared to the same dimer observed in CD27L. There is no cysteine present in the CTP1L side-by-side dimer interface, and the residue that is closest positioned is threonine T221 (Figure 2B). We mutated threonine T221 to a cysteine as well as an arginine, to see if we could emulate the effects observed for the C238R mutant in CD27L. We observed that both the T221C and the T221R mutant reduce autocleavage to almost undetectable levels, and that these mutants have a stronger effect than the V195P mutant (Figure 3D). This provides further support for the role of an oligomeric switch in the autocleavage of these endolysins.

### Endolysin oligomerization in solution

**Figure 5:**
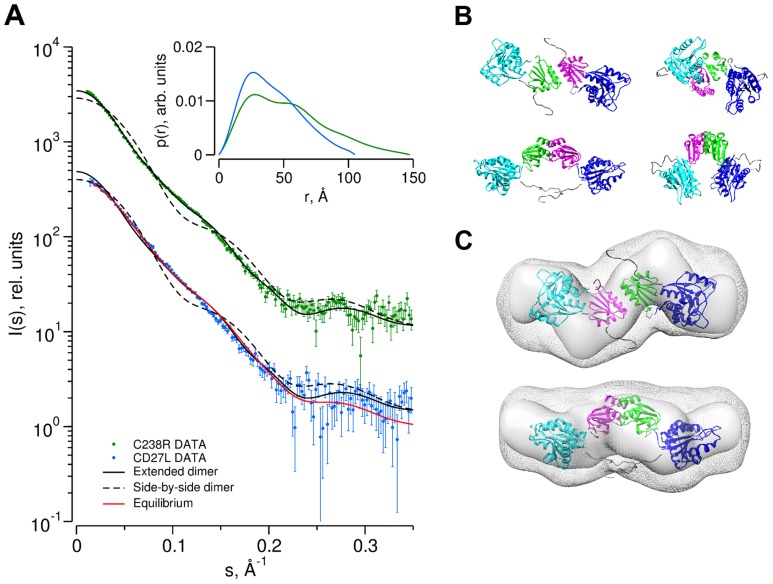
Determination of the dimer configuration of wild-type CD27L and mutant C238R in solution by SAXS. A. Overlay of the experimental scattering curves for wild-type CD27L (blue circles) and the C238R mutant (green circles) with the calculated scattering curves from a head-on-dimer/extended configuration (solid black line) and a side-by-side/compact dimer configuration (broken black line), built as a composite model from the crystal structures of the catalytic domain of CD27L (PDB code 3QAY) and the structure of the C-terminal domain presented in the paper, using the full length endolysin with a very similar catalytic domain PlyPSA amidase (PDB code 1XOV) to compose the full length structure. Missing regions of the structure (n-terminal histidine tag and interdomain linker residues) were refined against the SAXS data, keeping the domains fixed using the program CORAL. Also shown is the fit of the equilibrium model determined from OLIGOMER (solid red line) to the wild-type data. The inset shows the corresponding real-space distance-distribution functions *p(r)* determined by indirect Fourier transformation. B. Cartoon representations of the head-on dimer (left) and side-by-side dimer (right) configurations of CD27L C. Overlay of the reconstituted domains of CD27L C238R refined by rigid body modeling (cartoon) with the best and average SAXS bead models in surface and mesh presentation, respectively. At the center of the dimer sit two C-terminal domains in a head-on configuration. The catalytic domains are at the exterior, and the N-terminal His tag and inter-domain linker are shown as grey spheres. Models rotated by 90° are shown below each corresponding reference structure.

To determine the low resolution shape and the oligomeric state of CD27L endolysin in solution, small-angle X-ray scattering (SAXS) experiments were conducted using freshly purified material (Table 2). We used the crystal structures of the catalytic domain of CD27L (PDB code 3QAY) and the crystal structure of the C-terminal domain presented here to make a composite model of the full length CD27L endolysin using the structure of the intact PlyPSA amidase (PDB code 1XOV) to place the two domains. This model was employed to test the presence in solution of the two dimeric states of the C-terminal domain observed in the crystal structure. The molecular mass of the solute of wild type full length CD27L, estimated from the forward scattering intensity was 42±4 kDa, significantly lower than expected for a 64 kDa dimer and indicative of a possible equilibrium of the dimers with dissociation products. SAXS curves calculated from both the head-on and side-by-side dimers using CRYSOL [23] produced poor fits (discrepancy χ = 1.8 and 4.0) to the experimental data from the wide type protein (Figure 5A). For the C238R mutant, however, the experimental data fit a scattering curve calculated from the head-on dimer configuration of the composite model (discrepancy χ = 1.0) (Figure 5A). The side-by-side dimer is not compatible with the scattering curve for this mutant (χ = 3.3). This is an indication that this mutation has driven the equilibrium of the oligomeric states towards the head-on dimer. The distance distribution function *p(r)* of the C238R mutant (Figure 5A, insert) displays two distinct peaks, the one at larger distance (about 70 Å) matching the distance between the centers of the catalytic domains in the head-on dimer. The *p(r)* function of the wild type lacks this feature and displays a smaller maximum size, again suggesting an equilibrium of dimers and dissociation products.

**Table 2:** SAXS Data collection and derived parameters for CD27L.

	CD27L (wild-type)	CD27L (C238R)
**Data collection parameters**		
Instrument	EMBL X33 beam line (DORIS-III, DESY, Hamburg)	EMBL P12 beam line (PETRA-III, DESY, Hamburg)
Beam geometry	2.0×0.6 mm^2^	0.2×0.12 mm^2^
Wavelength (Å)	1.54	1.24
*s* range (Å^−1^)^a^	0.01–0.6	0.01–0.46
Exposure time (s)	8×15	1 (20×0.05 s)
Concentration range (mg/mL)	0.9–4.0	1.0–8.5
Temperature (K)	283	283
**Structural parameters** b		
*I(0)* (relative) [from *p(r)*]	44±2	3653±14
*R_g_* (Å) [from *p(r)*]	33±1	43±2
*I(0)* (cm^−1^) (from Guinier)	45.6±0.5	3664±14
*R_g_* (Å) (from Guinier)	33±1	42±1
*D_max_* (Å)	106	147
Porod volume estimate (Å^3^)	72151±10000	91690±10000
Excluded volume estimate (Å^3^)	94000±10000	123000±10000
Dry volume calculated from sequence (Å^3^)	39121/78219 (mon/dim)	
**Molecular-mass determination**		
*I(0)* (cm^−1^) BSA (66,000 Da)	71.4±0.4	3791±10
Molecular mass *M*_r_ (Da) [from *I(0)*]	42150±5000	63780±5000
Molecular mass *M*_r_ (Da) [from Porod volume (*V_p_/1.6)*]	45094±5000	57306±5000
Molecular mass *M*_r_ (Da) [from excluded volume (*V_ex_/2)*]	47000±5000	61500±5000
Calculated monomeric *M*_r_ from sequence	∼32335	
**Software employed**		
Primary data reduction	RADAVER	
Data processing	PRIMUS/Qt	
Ab initio analysis	DAMMIF	
Validation and averaging	DAMAVER	
Rigid-body modeling	CORAL	
Equilibrium analysis	OLIGOMER	
Computation of model intensities	CRYSOL	
3D graphics representations	PyMOL, UCSF Chimera	

Abbreviations: *M_r_*: molecular mass; *R_g_*: radius of gyration; *D_max_*: maximal particle dimension; *V_p_*: Porod volume; *V_ex_*: Particle excluded volume.

a Momentum transfer |*s*| = 4πsin(θ)/λ.

b Values reported for merged data sets (wild-type: 0.9 & 4.0 mg.mL^−1^, C238R: 1 & 8.4 mg.mL^−1^).

Low resolution shape reconstruction from the SAXS data for the wild-type and C238R mutant yields compact and extended structures, respectively. These models represent an average of the conformations of all particles present in solution. The volumes of the models constructed from the wild-type (*V_ex_* = 94000±10000 Å^3^) and C238R (*V_ex_* = 123000±10000 Å^3^) data are consistent with that of a mixture and of a dimeric CD27L structure, respectively (Table 2). The extended shape reconstructed for the C238R mutant overlays well with the head-on dimer model, providing a good low resolution representation of the solution structure (Figure 5C).

**Figure 6:**
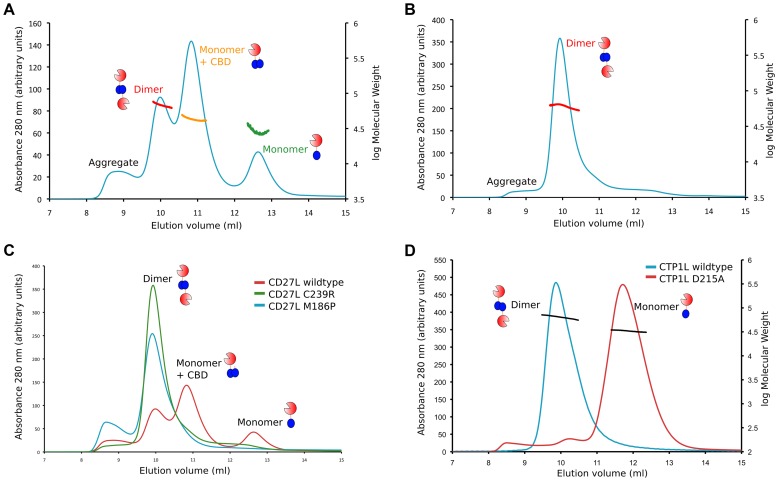
Oligomerization of CD27L in solution for wild-type and the M186P and C238R mutants. A. Size exclusion chromatography elution profile of the UV trace at 280-type CD27L. There are three peaks that correspond to the dimer (68±4 kDa), a mixture of monomer and cleaved C-terminal domain (43±2 kDa), and the monomer (33±7 kDa). B. Elution profile of the CD27L C238R mutant, showing a single peak with a dimeric species (62 kDa). C. Superposition of the elution profiles of the wild-type and the C238R and M186P mutants, showing the relative peak heights at equivalent amounts of sample loaded, with increased aggregation for the M186P mutant. D. Superposition of the elution profiles of wild-type CTP1L (in blue) and the CTP1L mutant D215A (in red).

We then performed gel exclusion chromatography coupled with right-angle light scattering and refractive index/UV measurements to assess the molecular weights of each endolysin species, comparing the separation profiles of the wild type CD27L with the C238R and M186P mutants. The elution profile of the wild type CD27L endolysin is rather complicated (Figure 6A). We interpret the peak with a molecular weight mass of 68±4 kDa as predominantly containing endolysin dimers (expected *MW* is 64 kDa), and the peak with a molecular weight mass of 33±7 as a CD27L monomer. In between, there is a peak at 43±2 kDa molecular weight that we interpret as a mixture of CBD-cleaved monomer in complex with full length protein (expected *MW*, 42 kDa). We conclude that the wild-type protein exists in different oligomeric states in solution that are affected by autoproteolytic cleavage. The C238R mutation produces an elution profile with a single peak corresponding to the MW of a dimer (61±3 kDa) (Figure 6B). The M186P mutant also appears to exist predominantly as a dimer (MW, 63±3 kDa, Figure 6C), but it has a tendency to form aggregates. It is interesting to note that both mutations force the CD27L endolysin to adopt a dimeric state, even though only one mutation, M186P, is incorporated directly at the autoproteolytic cleavage site. A more distinct elution profile is obtained for wild-type CTP1L, which exclusively forms a dimer with an average molecular weight of 66±5 kDa (Figure 6D). A CTP1L mutant in the head-on dimer interface (D215A) that reduces cleavage behaves predominantly as a monomer (average molecular weight 33±1 kDa), with a small portion present as a dimer.

The results of the size exclusion analysis of CD27L wild-type and mutants suggest that both the integrity of the internal cleavage site combined with how endolysin self-associates are key factors that dictate the final auto-cleavage event. M186P abolishes cleavage, as indicated by the disappearance of the intermediate 43 kDa species as well as the monomer peak from the elution profile. Abolishing side-by-side dimer formation via the introduction of a C238R mutation produces a dimeric state that is less-prone to aggregation. In addition, autoproteolytic cleavage has ceased, and the sole presence of the head-on dimer leads to an elution profile with a single dimer peak. Consequently, auto-proteolytic cleavage appears to be a spatially controlled *trans* event that occurs between endolysin monomers but only when these monomers associate to adopt the appropriate dimeric – or oligomeric – state.

To further investigate oligomerisation states and potential degradation of the CD27L samples in solution, the experimental data was analyzed in terms of possible mixtures using OLIGOMER [24]. The extended head-on and compact side-by-side dimer models and their individual domains were used to generate form-factor files for a fitting procedure, where volume fractions of each component present were determined that minimize the discrepancy between the theoretical scattering of the components and the experimental data (Table 3). The contribution from the potential degraded products including the lysed side-by-side dimer with a missing catalytic domain, dimers of C-terminal domains and the individual domains were pooled together as an additional component. The C238R data is described exclusively by the extended head-on dimer component scattering. The head-on dimer is also the dominant component in solution for wild-type, but the other components show noticeable contributions providing the best description of the wild-type data. This result further explains the low apparent molecular mass determined from the wild-type SAXS data, and also why the individual structures show such poor fits to the wild-type data (Figure 5A).

**Table 3:** Equilibrium analysis of the SAXS data using the program OLIGOMER.

Sample^a^	Volume Fractions			Fit, χ
	Extended Dimer	Compact Dimer	Degradation Products	
CD27L	0.41±0.02	0.24±0.02	0.35±0.02	1.5
C238R	0.98±0.01	0.02±0.01	0.0	1.1

a Values reported for merged data sets (wild-type: 0.9 & 4.0 mg/mL, C238R: 1 & 8.4 mg/mL).

### Specific cross-linking confirms existence of head-on dimer in the CTP1L endolysin

**Figure 7:**
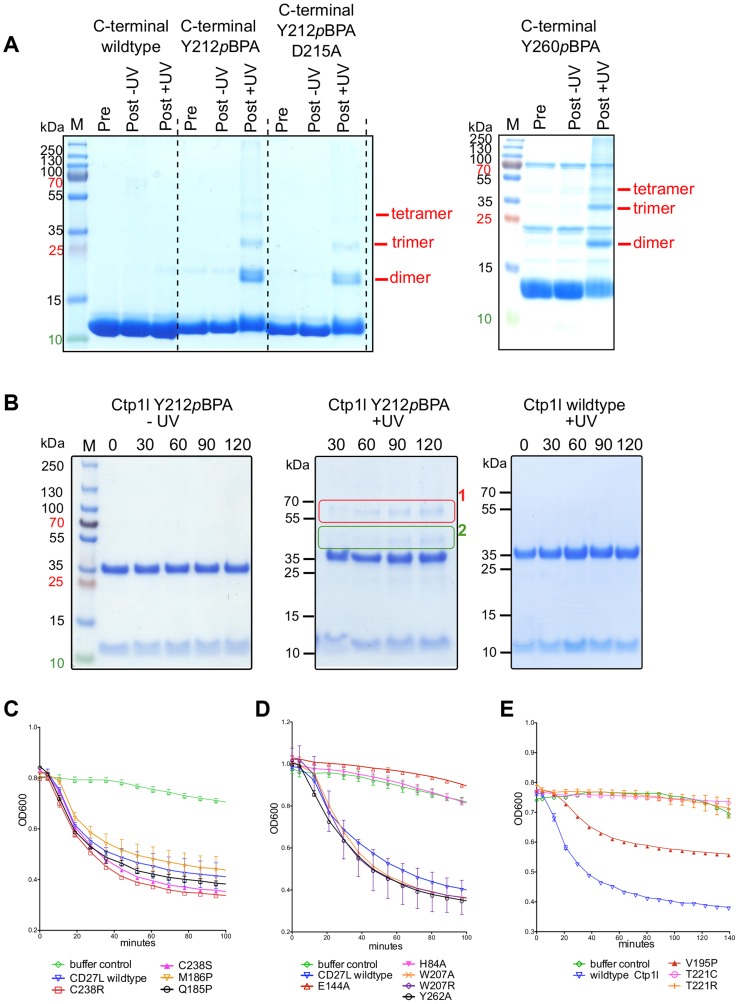
Cross-linking of oligomers and lytic activity of endolysins CD27L and CTP1L. A. SDS-PAGE of the C-terminal domain of wild-type CTP1L with the light sensitive cross-linker pBpa incorporated at positions Y212 or Y260 respectively, as well as the double mutant Y212pBpa/D215A. B. SDS-PAGE of the full-length endolysin with the Y212pBpa cross-linker mutant, showing the presence of 1) a full-length dimer, 2) an oligomer consisting of one C-terminal domain and a full-length CTP1L endolysin molecule. C, D. Lysis assays of 10 µg of recombinant CD27L applied to a culture of *C. difficile* showing that the autocleavage mutants do not affect lysis *in vitro* and that amidase active site mutants are not active. E. Lysis assays of CTP1L on *C. tyrobutyricum* cells showing the effect of mutants that reduce autocleavage (V195P, T221C and T221R).

To independently verify oligomerization of the CTP1L endolysin, we cloned the C-terminal domain alone and expressed it in *E. coli*. We introduced an amber stop codon at position Y212 which sits on alpha helix α1 (Y212pBpa). We also introduced an amber stop codon at position Y260, which is situated on alpha helix α3 (Y260pBpa). Both alpha helix α1 and α3 are involved in head-on dimerization (Figure 2B and 4A, B). We then expressed both amber mutants in the presence of the cross-linkable amino acid p-benzoyl-L-phenylalanine (pBpa) together with a pBpa specific tRNA and a tRNA synthetase that are capable of placing the pBpa at the position of the amber stop codon. In this way, a light sensitive cross-linker is introduced with a specific radius of interaction of approximately 10 Ångstrom [25]. The incorporation of the unnatural amino acid was confirmed for both mutants by tryptic digest, followed by mass spectrometry. We show that upon exposure to UV light, both the Y212pBpa and the Y260pBpa mutants show an additional band on an SDS-PAGE gel at double the molecular weight of the C-terminal domain alone (Figure 7A), whereas the unexposed and the wild-type protein do not show any cross-linked material. A mutant (D215A) that affects the head-on dimer in CTP1L does also cross-link when combined with the Y212pBpa mutant, indicating that the cross-linking process captures transient oligomeric states over an extended period of time (2 hours). The band with elevated molecular weight was treated with trypsin and analyzed by mass spectrometry and it was confirmed that it contained the C-terminal domain of CTP1L. We also see faint larger bands that could consist of the trimer and tetramer. Since the pBpa cross-linking is quite specific, we conclude that the head-on dimer is also formed by the C-terminal domain of CTP1L in solution. Finally, we introduced an amber stop codon in the full length CTP1L endolysin replacing Y212, which showed a higher final yield of pBPA incorporation when compared to position Y260. The full length CTP1L protein is cross-linked only upon exposure to UV light and forms a mixture of full length CTP1L dimers, as well as a species that based on the molecular weight consists of one full length CTP1L and a C-terminal domain fragment (Figure 7B). The oligomerization states of the CTP1L fragments observed by cross-linking reinforce the interpretation of the size exclusion chromatography and light scattering experiments done on the CD27L endolysin, showing that both the cleavage and the oligomerization occur in both endolysins.

### Inhibition of autoproteolytic cleavage inactivates the related endolysin CTP1L

To verify whether the autoproteolytic cleavage affects the activity of the endolysins containing the C-terminal domain, we performed cell lysis on *C. difficile* cultures with recombinant CD27L wild type and mutants using turbidity reduction assays (Figure 7C, D). We observed no difference in lysis efficiency between the wild type protein and mutants which prevent/reduce cleavage either at the cleavage site (M186P) or by affecting the side-by side (C238R and C238S) or head-on dimers (W207A, W207R and Y262A). This establishes at the least that these mutants are enzymatically active, but it does not resolve whether autocleavage plays a role in endolysin function. Introduction of mutations at the catalytic site (H84A and E144A) did abolish lytic activity (Figure 7D) but did not affect cleavage (Figure 3B). It was previously established that when applied externally to *C difficile* cells, a construct that contains the enzymatic domain alone shows the same lytic efficiency as the full length protein [7]. Therefore, the lysis assay is insensitive to the trigger and release function of the C-terminal domain for CD27L.

However, CTP1L is only active as an intact, full length protein, and the enzymatic domain alone does not lyse *C. tyrobutyricum* cultures [14]. Lysis of *C. tyrobutyricum* cultures by wild-type CTP1L is robust (Figure 7D), leading to a drop in optical density (OD) at 600 nm. The mutants show a drop in lysis efficiency that is proportional to the reduction in autocleavage. The V195P mutant is still somewhat active, whereas the T221R and T221C mutants show no lysis at all. We verified that these mutants are similar in secondary structure to the wild-type (Figure S1). We conclude that in the context of an externally applied recombinant endolysin, CTP1L depends on the autoproteolytic cleavage of its C-terminal domain to lyse *C. tyrobutyricum*.

## Discussion

Bacteriophages release endolysins at the end of the phage life cycle to lyse the host bacterial cell following a well-timed trigger mechanism [9]. The molecular mechanisms underlying such a trigger are unknown, but it is thought that endolysins are activated after the formation of holin lesions in the bacterial cell membrane [10]. When the endolysins pass from the cytosol to the extra-cellular environment, they will undergo a substantial change in environment and this may activate the endolysin.

The crystal structures presented here for the C-terminal domains of two endolysins that target *Clostridia* bacteria (CD27L and CTP1L) suggest that CD27L exists in two distinct dimeric states. We show indirectly that these dimeric states are associated with an autocleavage mechanism, because several mutations in the dimer interfaces reduce autocleavage. Endolysin dimerization has been shown for other bacteriophage species, and the dimerization seems to influence endolysin activity. The pneumococcal autolysin LytA does dimerize into a conformation resembling the side-by-side dimer presented here, and it was suggested that the dimer conformation may contribute to its activity [26]. The CPL-1 phage endolysin that targets *Streptococcus pneumoniae* was engineered to stabilize the (what we call) side-by-side dimerization mode, and this led to a ten fold increase in its activity [27].

We propose that the oligomeric switch can be described in terms of an Monod-Wyman-Changeux mechanism [28], with a ‘tensed’ state that represents the inactive endolysin and a ‘relaxed’ state that represents the active endolysin. We propose that the ‘tensed’ state is related to the head-on dimer, where the two endolysin units are extended and the two autocleavage sites are far apart. The ‘relaxed’ state is the side-by-side dimer, which promotes autocleavage and the release of the catalytic domain from the C-terminal domain. Autocleavage increases the action radius of the catalytic module, and as previously suggested [29], the small globular size of this enzyme may allow it to further penetrate the bacterial cell wall which may act as a sieve. Bacteriophages have been shown to use a mechanism of autocleavage and oligomerization when entering the bacterial cell wall upon infection [30]. Some bacterial toxins are activated upon autocleavage [31], [32]. We have not been able to identify residues that catalyze the cleavage of the catalytic domain, but we managed to switch the cleavage off with a point mutation (M186P) at the hinge of the linker. The presence of a methionine at this position for CD27L seems to be of significance, as can be seen from the sequence alignment between lysins with a similar domain (Figure 2A). According to the sequence alignment presented in Figure 2, all lysins that have a cysteine present at position 238, also have a methionine at the start of the domain. It is interesting to note in this respect that a chimera between the catalytic domain of CS74L and the C-terminal domain of CD27L (CS74L_1-177_-CD27L_180-270_) [33] also cleaves off its C-terminal domain (unpublished results). The C-terminal domain could therefore be involved in autonomous self-cleavage, but this needs to be further investigated.

At this stage, we can only speculate about the role of the side-by-side dimer in the autocleavage mechanism. We believe that this dimerization mode will affect the conformation of the linker that connects the two domains, possibly bringing two linkers within close proximity. The methionine M186 of CD27L and the valine V195 of CTP1L may be involved in cis- (within the linker itself) or trans (in an exchange between the two linkers) autoproteolysis, such as is observed for other bacterial enzymes that undergo maturation [34]. This would represent a new form of protein splicing, involving two copies of the endolysin, rather than a single autonomous splicing unit such as is observed in inteins [35]. We are in the process of further investigating this splicing mechanism.

We have shown that autocleavage is an intrinsic property of two endolysins targeting Clostridia, and we believe that this mechanism occurs in other endolysin systems as well. The most potent lysin identified to date (PlyC) consists of two components that are expressed independently [16]. Structural characterization revealed that one component provides dual catalytic activity, whereas the other component is an octomeric cell wall binding unit. The lack of a covalent link between the enzymatic portion and the cell wall binding domain is probably key for its increased potency. We therefore believe that the engineered clustering of endolysins through a controlled oligomerization of the C-terminal domains may lead to more efficient enzymes with high specificity. This opens new opportunities to produce recombinant phage or endolysins that can lyse specifically pathogenic bacteria without affecting the microbiome overall.

## Materials and Methods

### Protein expression, purification and crystallization

The nucleotide sequence of the full-length endolysins CD27L and CTP1L mutant V195P, as well as the C-terminal domain CD27L_180-270_ were inserted in pET15b, containing an N-terminal His tag and a thrombin cleavage site as described previously [7]. Constructs were expressed in *E. coli* BL21(DE3) grown in Lysogeny broth (LB) media until an OD_600_∼0.6 was induced with 1 mM isopropyl-β-D-thio-galactopyranoside for overnight expression at 21°C. Protein expressing cells were harvested by centrifugation (5500 rpm, 30 min) and the supernatant discarded. Pelleted cells were lysed chemically in lysis buffer (50 mM Tris pH 8.0, 300 mM NaCl, 1% Triton X-100, 10 mM Imidazole, 1 mg/ml Lysozyme, 25 U/ml Benzonase nuclease) for 30 min at 4°C. Lysed cell extract was centrifuged (18,000 rpm, 40 min) and supernatant containing His-tagged endolysin purified by nickel-nitrilotriacetic acid (Ni-NTA) purification (Qiagen). Protein was eluted in a final elution buffer of 50 mM Tris pH 8.0, 150 mM NaCl, 200 mM Imidazole. Proteins were purified for crystallization by size exclusion chromatography using an Aekta liquid chromatography system (Amersham Biosciences) and S75 10/300 GL (Tricorn) column (GE Healthcare) in 20 mM HEPES, pH 7.4. The protein was concentrated to 10 mg/mL as measured by UV absorption at 280 nm. Protein crystals for degraded CD27L, that ultimately only contained the C-terminal domain, were obtained from a mother liquor containing 10 – 20% PEG 4000 and 20 mM Tris pH 8.0. Crystals of the construct containing the C-terminal domain of CD27L and an N-terminal His tag were obtained from a mother liquor of 10% PEG 20K and 20 mM Tris pH 8.0. For the CTP1L V195P mutant, crystals were obtained from a mother liquor containing 20 mM TRIS pH 8.0 and 6% PEG 8000.

### Crystal structure determination of CD27L

The C-terminal domain of CD27L was first solved by single-wavelength anomalous dispersion using a mercury derivative (Table 1). Crystals of the CD27L C-terminal domain construct alone with an N-terminal His tag were soaked in a cryo-protecting solution containing 15% PEG 20K, 100 mM Tris pH 8, 10% (v/v) glycerol and the derivative 1 mM of Ethyl-mercury phosphate for a few minutes prior to freezing. A data set was collected on the X12 beamline at EMBL Hamburg, which is equipped with a MAR225 CCD detector. The crystal diffracted to a resolution of 3.5 Å, and the space group was P2_1_. All the X-ray data were indexed, merged and scaled with DENZO and Scalepack [36]. The crystal contained eight copies of the C-terminal domain in the asymmetric unit, and 8 mercury sites were identified with SHELXD [37]. Density modification was performed with PARROT, and an initial model was built with BUCCANEER [38]. This model was used in PHASER [39] to further improve the experimental phases and to find 5 additional mercury sites after several iterations.

A native X-ray data set was collected on PROXIMA I at the Soleil Synchrotron (Gif-sur-Yvette, France), using a Q315 CCD detector from ADSC. The crystal diffracted to 2.3 Å and belonged to space group P2_1_2_1_2_1_. The initial model was then used in molecular replacement using MOLREP [40] to identify the contents of the crystals grown from initial full length CD27L. It was determined that these crystals contained six copies of the C-terminal domain in the asymmetric unit. The structure was refined with Refmac5 [41] to an R factor of 19.8% (Rfree  = 25.6%). The stereochemistry of the model contained 98.2% of the residues within the favored areas of the Ramachandran plot according to Molprobity [42], and no residues in the disallowed regions.

### Crystal structure determination of the C-terminal domain of CTP1L mutant V195P

A native X-ray data set was collected on the EMBL beamline P14 at the PETRA3 synchrotron (Hamburg, Germany) using a MAR225 CCD detector. Although the crystal probably diffracted to at least 1.5 Ångstrom resolution, we were only able to collect usable data to a resolution of 2.1 Ångstrom due to a limited detector geometry. The crystal diffraction also suffered from ice rings, limiting the completeness of the data to 92%. Nevertheless, it was straightforward to solve the structure of the C-terminal domain of CTP1L by molecular replacement with MOLREP [40] using the C-terminal domain of CD27L as a search model, since there is only one copy of the molecule in the asymmetric unit. The structure was refined with Refmac5 to an R factor of 17.2% (Rfree  = 26.4%), and the electron density is of good quality. The stereochemistry of the refined model contained 98.8% of the residues within the favored areas of the Ramachandran plot according to Molprobity [42], and no residues in the disallowed regions.

### Mutagenesis of CD27L and CTP1L mutants

The mutants of CD27L and CTP1L were created by PCR site-directed mutagenesis following the Quikchange method (Stratagene). Plasmids pET15b*-cd27l* [5] and pET15b-*ctp1l* [14] were used as template DNA. Complementary primer pairs for each mutation (Table S1) were used for whole plasmid mutagenesis PCR performed using Phusion polymerase (NEB). Template DNA was digested by *Dpn*I (NEB) before transformation into competent *E. coli* DH5α (Invitrogen). Plasmid DNA was obtained by Miniprep (Qiagen) for sequence confirmation. Mutants were expressed and purified using the same method as wild-type CD27L.

### SDS-PAGE analysis

Samples of all constructs were mixed with reducing Laemmli buffer, heated for 5 minutes at 75°C and subjected to 15% SDS polyacrylamide gel electrophoresis. For Coomassie Blue staining, the SDS-PAGE gel was incubated respectively in Coomassie Blue staining solution (0.125% Coomassie Blue, 45% ethanol, 10% acetic acid), destaining solution (40% ethanol, 10% acetic acid) and drying solution (2% glycerol, 20% ethanol).

### Intact protein sample analysis by LC-MS

Protein samples (around 2 mg/mL) were acidified using 1% formic acid solution and transferred to vials prior to LC-MS analysis. Desalting and protein separation were carried out using an Acquity UPLC system (Waters) fitted with a C_4_ column (2.1 mm×15 cm, 5 µm particle size). The column was maintained at constant temperature (40°C) throughout. The outlet of the column was coupled directly to a Q-Tof II mass spectrometer (Waters) using the standard ESI source in positive ion mode.

Solvent A was water, 0.1% formic acid and solvent B was acetonitrile, 0.1% formic acid. The samples (between 1 and 20 µL) were loaded onto the column and desalted for 5 minutes at a flow rate of 0.2 mL/min, 4% B. The proteins were then eluted from the column with a constant flow of 0.2 mL/min. During the elution step, the percentage of solvent B increased in a linear fashion from 5% to 25% in 1 minute, then increased to 80% in a further 11 minutes. On the Q-Tof, a spray voltage of 3.5 kV was applied, with a cone voltage of 35 V and extraction cone at 10 V. A collision energy of 8 eV was used, with Argon in the collision cell. The desolvation temperature was set at 320°C, with a source temperature of 120°C. Data were acquired in continuum mode, over a mass range 500-3500 *m/z* with a scan time of 0.5 s and interscan delay of 0.1 s. Data were externally calibrated against a reference standard of intact myoglobin, acquired immediately after sample data acquisition. Spectra across the protein chromatographic peak(s) were summed and intact mass was calculated using the MaxEnt1 maximum entropy algorithm (Waters/Micromass) to give the zero charge deconvoluted molecular weight.

### SEC-RALS/RI/UV molecular weight determination

Molecular weight estimates of several CD27L variants (CD27L wild-type, CD27L C239R and CD27L M186P) as well as CTP1L and CTP1L D215A were evaluated using size-exclusion chromatography in combination with right-angle light scattering (RALS), refractive index (RI) and UV (λ_280 nm_) measurements (Malvern Instruments Viscotek, RALS/RI/UV 305 TDA detector equipped with a 670 nm laser diode). All measurements were performed at room temperature. Samples were separately injected at their respective concentrations (75 µL at 6.37, 7.15 and 6.17 mg.mL^−1^) onto a GE-Healthcare Tricorn S75 10/300 GL column equilibrated in 20 mM HEPES pH 7.4, 500 mM NaCl at a flow rate of 0.4 mL.min^−1^. The molecular weight (*MW*) of each species eluting from the SEC column were assessed using concentration (*c*) measurements derived from base-line corrected RI or UV measurements in combination with base-line corrected RALS intensities calibrated against a bovine serum albumin narrow (monomeric) standard (RALS  =  *c*(dn/dc)^2^.*MW*.k_RALS_; RI  =  *c*(dn/dc)k_RI_ and; UV  =  *cε*k_UV_, where dn/dc is the refractive index increment of unmodified protein, 0.185 mL.g^−1^, k_RI_, k_UV_ and k_RALS_ are the TDA instrument calibration constants relative to a BSA and ε the λ_280 nm_ E_0.1%_ extinction coefficient of each protein in mg.mL^−1^). The *MW* correlations across the selected range of each CD27L elution peak and the final *MW* estimates quoted in the text were calculated using OmniSEC Software (Malvern Instruments).

### SAXS data collection and shape determination

Synchrotron radiation X-ray scattering data were collected on the EMBL X33 and P12 beamlines of the storage rings DORIS III and PETRA III (DESY, Hamburg), respectively, using PILATUS 1 M and 2 M pixel detectors (DECTRIS, Switzerland). For the wild-type CD27L data were acquired at X33, with 8 frames of 15 s exposure time collected. Samples were measured in a temperature controlled cell at 10°C in 20 mM HEPES buffer, 150 mM NaCl pH 7.4 at protein concentrations of 0.9–4.0 mg/mL. The sample-to-detector distance was 2.7 m, covering a range of momentum transfer 0.01≤s≥0.6 Å^−1^ (s = 4π sinθ/λ, where 2θ is the scattering angle, and λ = 1.54 Å is the X-ray wavelength). For the C238R mutant data were acquired at P12, with 20 frames of 0.05 s exposure time collected. Solutions were measured while flowing through a temperature controlled capillary at 10°C in 20 mM Tris buffer, 500 mM NaCl pH 7.4 at protein concentrations of 1.0–8.5 mg/mL. The sample-to-detector distance was 3.1 m, covering a range of momentum transfer 0.008≤s≥0.458 Å^−1^ (s = 4π sinθ/λ, where 2θ is the scattering angle, and λ = 1.24 Å is the X-ray wavelength). Based on comparison of successive frames, no detectable radiation damage was observed. Data from the detector were normalised to the transmitted beam intensity, averaged and the scattering of buffer solutions subtracted. The difference curves were scaled for solute concentration and the 1.0 mg/mL (low-*s*) and 8.4 mg/mL (high-*s*) data sets merged for modeling. All data manipulations were performed using PRIMUS [43].

The forward scattering *I(0)* and radius of gyration, *Rg* were determined from Guinier analysis [44], assuming that at very small angles (s≤1.3/*Rg*) the intensity is represented as *I(s) = I(0)*exp(-(*sRg*)^2^/3)). These parameters were also estimated from the full scattering curves using the indirect Fourier transform method implemented in the program GNOM [45], along with the distance distribution function *p(r)* and the maximum particle dimension *D_max_*. Molecular masses (MMs) of solutes were estimated from SAXS data by comparing the extrapolated forward scattering with that of a reference solution of bovine serum albumin, and also from the hydrated-particle/Porod volume *V_p_*, where molecular mass is estimated as 0.625 times *V_p_*.

### Ab initio shape determination and molecular modelling

Low-resolution shape envelopes for all constructs were determined using the *ab initio* bead-modelling program DAMMIF [46], using both P1 and P2 symmetry. The results of 10 independent DAMMIF runs were analyzed using the program DAMAVER [47] to identify the most representative/typical models. Modeling using P2 symmetry was only attempted following the identification of excluded solvent volumes, *V_ex_* in models generated in P1 (slow mode) consistent with that expected for dimers (see Table 2).

Molecular modelling was conducted using, as rigid bodies and where appropriate, the crystal structures of the catalytic and the C-terminal domains of CD27L determined in this study. Rigid-body models were generated using the program CORAL [24] and 10 independent runs assessed for convergence with DAMAVER. Additional fitting of PDB files to the SAXS data was conducted using CRYSOL [23].

### Oligomeric equilibrium analysis

Using the program OLIGOMER [24], the SAXS data for both wild-type CD27L and the C238R mutant was used to model potential multicomponent mixtures of species in solution. Form factors of input PDB files were calculated using the program FFMAKER [24]. Form factors were also calculated for individual domains and substructures of the intact PDB files to represent products of autolysis and averaged. This averaging was performed as the identity of the exact solution composition of these lysis products could not be established. Volume fractions corresponding to each component (eg. extended dimer, compact dimer and “degraded components”) were determined by OLIGOMER utilising a non-negative least squares procedure.

### Sub-cloning and expression for *p*-benzoyl-L-phenylalanine incorporation into CTP1L

Following the principal method outlined by Farrell *et al*. [48], the photo-activated amino acid *p*-benzoyl-L-phenylalanine (*p*BPA) (BACHEM) was incorporated into the full-length CTP1L endolysin and the truncated C-terminal domain of CTP1L.

CTP1L was amplified from *ctp1l*-pET15b [14] and inserted into the pET21d vector. The *amber* codon (TAG) was incorporated at position Y212 or Y260 using complementary primer pairs (Table S1) following the Quikchange method of PCR site-directed mutagenesis to generate *Y260TAG*-pET21d and *Y212TAG*-pET21d. Sub-cloning into pET21d introduced a C-terminal hexa histidine-tag to the construct, which permits selective Ni-NTA purification of full-length proteins that have only incorporated *p*BPA. C-terminal constructs containing Y260TAG and Y212TAG were generated by amplifying the C-terminal domains of *Y212TAG*-pET21d and *Y260TAG*-pET21d between positions V195 and K274. The PCR products were inserted back into the pET21d vector to create *C-termY260TAG*-pET21d and *C-termY212TAG-*pET21d. As a control, the wild-type C-terminal domain was sub cloned into pET21d with no amber stop codon incorporated. The C-terminal domain double mutant combining Y212TAG and D215A was created with complementary primers containing both mutations and using the wild-type C-terminal domain construct as a template.

*E.coli* BL21(AI) cells were transformed with pEVOL-*p*BPA (aminoacyl-tRNA synthetase/suppressor tRNA) and one of the plasmids encoding an *amber* codon containing construct: *Y212TAG-*pET21d, C-term*Y212TAG*-pET21d or *C-termY260TAG-*pET21d. Cells were grown in 500 ml Lysogeny broth (LB) media supplemented with 1 mM *p*BPA in the presence of ampicillin and chloramphenicol. When an OD_600_ of 0.6 had been reached the cultures were induced with Arabinose (final concentration 0.02%) and expressed at 21°C overnight. Cells were harvested by centrifugation (5500 rpm, 30 min) and the supernatant discarded.

### Photo-cross-linking full length CTP1L and the C-terminal domain of CTP1L

The full length CTP1L mutant Y212*pBPA* was Ni-NTA purified as described above and dialyzed into 25 mM TRIS, pH 7.4. The protein was concentrated to 0.5 mg/mL, as measured by UV absorption at 280 nm. A 1 ml aliquot of purified protein was pipetted into a single well of a 24-well clear polystyrene plate, typically used for protein crystallography. The lid was kept on to prevent sample evaporation, and placed inside an RPR-100 UV reactor equipped with 350 nm bulbs (Rayonet). The reactor was kept at 4°C with the cooling fan on. Proteins were exposed to UV light for 15 minute intervals during which the solutions were stirred by gentle pipetting and samples taken for each time point for SDS-PAGE analysis. As a control, samples were also taken every 15 minutes from a parallel sample of Y212*p*BPA that was kept at 4°C in the dark with no UV exposure. Cross-linking was analyzed by SDS-PAGE by comparison of the pre-UV and the final post-UV exposed samples. The same photo-cross-linking experiment was performed for the C-terminal domain constructs. Ni-NTA purified C-termY212*p*BPA, C-termY260*p*BPA, C-termY212*p*BPA_D215A and C-terminal wild-type were dialyzed into 25 mM TRIS, pH 7.4 and concentrated to 2 mg/mL as measured by UV absorption at 280 nm. Following the same protocol as described above for the full-length Y212*p*BPA, 500 µl aliquots of each protein were pipetted into separate wells of a 24 well clear polystyrene plate and exposed to UV light for 120 minutes with stirring of the samples at 15 minute intervals. As a control SDS-PAGE samples were taken from parallel aliquots of each protein kept at 4°C in the dark with no UV exposure.

### Turbidity reduction assays

Lysis assays were performed on fresh cells of *C. difficile* NCTC 11204 or *C. tyrobutyricum* NCIMB 9582. Cells were cultured, harvested and resuspended in PBS pH 7.3 as described previously [5], [14]. Lysis assays were performed on freshly harvested cells in 300 µl volumes with 10 µg Ni-NTA-purified protein or elution buffer. Results are the mean of duplicate assays +/− standard deviation.

## Supporting Information

Figure S1 **Circular dichroism measurements on CD27L/CTP1L wild-type and mutant proteins to show integrity of secondary structure.** The mean residue molar ellipticity measure by CD is shown for (A) CD27L wild-type and the mutants that prevent autoproteolytic cleavage (M186P, W207A and C238R), and (B) CTP1L wild-type and the mutants that prevent autoproteolytic cleavage (V195P, T221C and T221R).
(PDF)

Methods S1 **CD spectropolarimetry measurements on CD27L and CTP1L endolysins and mutants.**
(DOC)

Table S1 **Primer pairs used for PCR site-directed mutagenesis of CD27L and CTP1L.**
(DOC)
