# Supplementary material for: The CD27L and CTP1L Endolysins Targeting Clostridia Contain a Built-in Trigger and Release Factor
Source: PLoS Pathog. 2014 Jul 24;10(7):e1004228. doi: 10.1371/journal.ppat.1004228 (PMC4110038; doi:10.1371/journal.ppat.1004228)

Figure S1: Circular dichroism measurements on CD27L/CTP1L wild-type and mutant proteins to show integrity of secondary structure

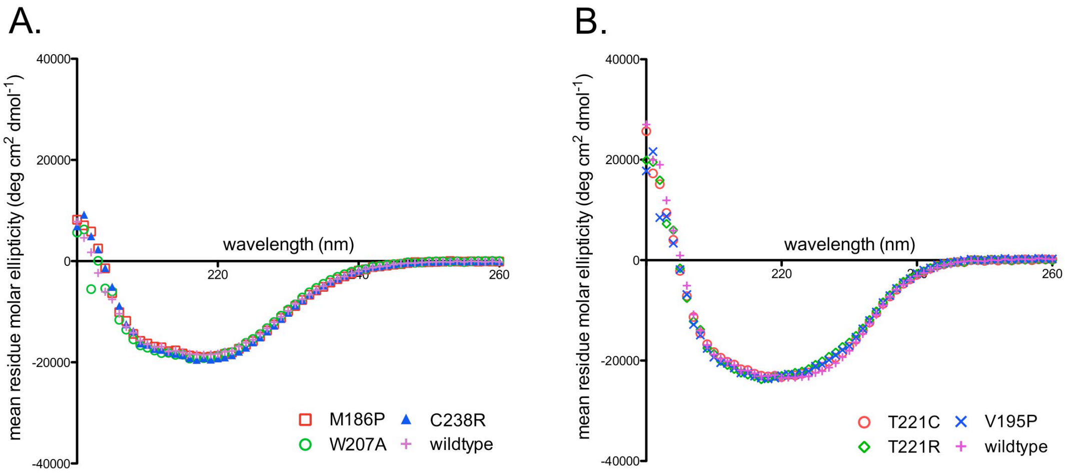

Supplement: Figure S1 — Circular dichroism measurements on CD27L/CTP1L wild-type and mutant proteins to show integrity of secondary structure. The mean residue molar ellipticity measure by CD is shown for (A) CD27L wild-type and the mutants that prevent autoproteolytic cleavage (M186P, W207A and C238R), and (B) CTP1L wild-type and the mutants that prevent autoproteolytic cleavage (V195P, T221C and T221R). (PDF) [file ppat.1004228.s001.pdf]
