# Supplementary material for: The CD27L and CTP1L Endolysins Targeting Clostridia Contain a Built-in Trigger and Release Factor
Source: PLoS Pathog. 2014 Jul 24;10(7):e1004228. doi: 10.1371/journal.ppat.1004228 (PMC4110038; doi:10.1371/journal.ppat.1004228)
Supplement: Methods S1 — CD spectropolarimetry measurements on CD27L and CTP1L endolysins and mutants. (DOC) [file ppat.1004228.s002.doc]

**Methods S1**

**CD spectropolarimetry measurements on CD27L and CTP1L endolysins and mutants**

Proteins were purified by Ni-NTA affinity purification as described above and further purified by size exclusion chromatography using an Aekta liquid chromatography system (Amersham Biosciences) and S75 10/300 GL (Tricorn) column (GE Healthcare). Size exclusion buffer for CD27L and CTP1L mutants was 20 mM HEPES, pH 7.4 with 500 mM NaCl and for CTP1L and mutants 20 mM HEPES pH 7.4. Size excluded protein was collected and dialyzed against 20 mM HEPES pH 7.4. Protein concentration was measured by UV absorption at 280 nm. Spectra were recorded at 20°C on a Chirascan CD Spectrometer (Applied Photophysics), between 200 and 260 nm in a 0.1 cm cuvette. Machine settings were as follows: 1 nm bandwidth, 0.5-sec response, and 0.5-nm data pitch. Spectra were background-subtracted and converted into mean residue ellipticity. Each curve represents the mean of three separate measurements.
