# Supplementary material for: The CD27L and CTP1L Endolysins Targeting Clostridia Contain a Built-in Trigger and Release Factor
Source: PLoS Pathog. 2014 Jul 24;10(7):e1004228. doi: 10.1371/journal.ppat.1004228 (PMC4110038; doi:10.1371/journal.ppat.1004228)
Supplement: Table S1 — Primer pairs used for PCR site-directed mutagenesis of CD27L and CTP1L. (DOC) [file ppat.1004228.s003.doc]

**Table S1**: Primer pairs used for PCR site-directed mutagenesis of CD27L and CTP1L

| CD27L Mutation | Primer pair sequences |
| --- | --- |
| C238S | Forward 5’-GTTGTAGGAGGTGGCGCAAGTGAAAAGATAAGTTCTAT |
|  | Reverse 5’-ATAGAACTTATCTTTTCACTTGCGCCACCTCCTACAAC |
| C238R | Forward 5’-GTTGTAGGAGGTGGCGCACGTGAAAAGATAAGTTCTAT |
|  | Reverse 5’-ATAGAACTTATCTTTTCACGTGCGCCACCTCCTACAAC |
| M186P | Forward 5’-TAAAAATATAAATAATGAGGGAGTTAAACAGCCGTACAAACATACAATTGTTTATGATGGAGAA |
|  | Reverse 5’- TTCTCCATCATAAACAATTGTATGTTTGTACGGCTGTTTAACTCCCTCATTATTTATATTTTTA |
| Q185P | Forward 5’- TAAAAATATAAATAATGAGGGAGTTAAACCGATGTACAAACATACAATTGTTTATGATG |
|  | Reverse 5’- CATCATAAACAATTGTATGTTTGTACATCGGTTTAACTCCCTCATTATTTATATTTTTA |
| CTP1L Mutation |  |
| V195P | Forward 5’-GATGAATTTATAAAATATATTAAGGGGGAAGATGAACCGGAAAATTTAGTAGTTTATAATGATGG |
|  | Reverse 5’-CCATCATTATAAACTACTAAATTTTCCGGTTCATCTTCCCCCTTAATATATTTTATAAATTCATCC |
| T221R | Forward 5’-TTAGCAGATAGATTGGCATGTCCAAGGATTAACAATGCTAGGAAATTTGATT |
|  | Reverse 5’-AATCAAATTTCCTAGCATTGTTAATCCTTGGACATGCCAATCTATCTGCTAA |
| T221C | Forward 5’-TTTAGCAGATAGATTGGCATGTCCATGTATTAACAATGCTAGGAAATTTGAT |
|  | Reverse 5’- ATCAAATTTCCTAGCATTGTTAATACATGGACATGCCAATCTATCTGCTAAA |
| D215A | Forward 5’-CGGCAGAATATTTAGCAGCTAGATTGGCATGTCCAAC |
|  | Reverse 5’-GTTGGACATGCCAATCTAGCTGCTAAATATTCTGCCG |
| CTP1L pBPA incorporation |  |
| Y212TAG | Forward 5’-CAGACCAAAGAGCGGCAGAATAGTTAGCAGATAGATTGGCAT |
|  | Reverse 5’-ATGCCAATCTATCTGCTAACTATTCTGCCGCTCTTTGGTCTG |
| Y260TAG | Forward 5’-AATTGCAGGCTCAACAAGGTAGACAACTATGCAGGCAGTA |
|  | Reverse 5’-TACTGCCTGCATAGTTGTCTACCTTGTTGAGCCTGCAATT |
| Y212TAG_D215A | Forward 5’-CCAAAGAGCGGCAGAATAGTTAGCAGCTAGATTGG |
|  | Reverse 5’-CCAATCTAGCTGCTAACTATTCTGCCGCTCTTTGG |
